# Collective Variable-Guided Engineering of the Free-Energy Surface of a Small Peptide

**DOI:** 10.1021/acs.jcim.6c00674

**Published:** 2026-07-10

**Authors:** Muralika Medaparambath, Alexander Zhilkin, Dan Mendels

**Affiliations:** † The Wolfson Department of Chemical Engineering, 26747 Technion − Israel Institute of Technology, Haifa 32000, Israel; ‡ Faculty of Mathematics, Technion − Israel Institute of Technology, Haifa 32000, Israel

## Abstract

Engineering the free-energy surfaces (FES) of proteins and peptides is central to controlling conformational ensembles and their responses to perturbations. However, predicting how chemical modifications such as point mutations reshape the FES and shift conformational equilibria remains challenging, particularly in data-scarce settings. Building on the Collective Variables for Free-Energy Surface Tailoring (CV-FEST) framework, we develop a computational approach that leverages short, unbiased molecular dynamics trajectories to guide mutation analysis. Using the ten-residue β-hairpin CLN025 and a systematic library of its single-point mutants, we apply Harmonic Linear Discriminant Analysis (HLDA) to extract collective variables from the conformational data. We find that the HLDA eigenvector learned solely from short wild-type trajectories provides residue-level insights into the propensity of mutations at specific positions to thermodynamically stabilize or destabilize the folded state. Extending this analysis, we show that shifts in the leading HLDA eigenvalue across mutants, a measure of changes in separability between the conformational ensembles along the HLDA coordinate, correlate strongly with mutation-induced changes in the free-energy difference between states, as reflected in melting temperatures. Benchmarked against Replica-Exchange Molecular Dynamics simulations, these findings suggest a promising and computationally affordable route toward guiding the engineering of biomolecular free-energy landscapes.

## Graphical abstract

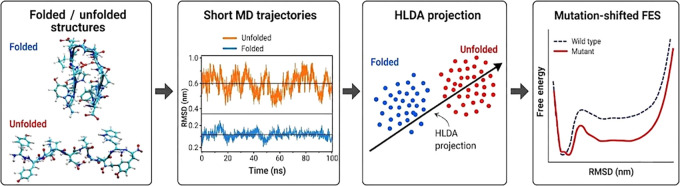

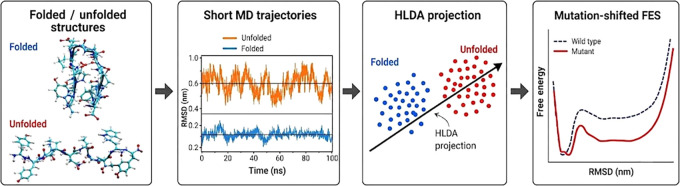

## Introduction

There has been remarkable progress in leveraging computational tools for protein engineering to accelerate research and development. This advancement has culminated in artificial intelligence (AI)-based structure prediction and generative models,^1–3^ most notably AlphaFold, which have transformed biological research. Yet, structure alone is not sufficient to explain protein function.^4^ Protein functionality is strongly influenced by thermodynamic and dynamic properties, or more broadly, by the features of the underlying free-energy surface (FES) and its response to perturbations such as mutations, coreceptor-induced structural changes, post-translational modifications, or environmental factors such as temperature and pH. This gives rise to a central challenge in biomolecular engineering: understanding and rationally modifying these FES, for example, through targeted mutations to existing proteins. Overcoming this barrier would unlock transformative applications, including designing proteins with tailored activities for targeted therapeutics,^5^ elucidating how mutant proteins contribute to cellular dysfunction and diseases, such as cancer,^6^ and engineering protein- or peptide-based materials.^7^

To this end, a substantial body of experimental and computational work has focused on predicting how point mutations affect native-state protein stability, motivated in part by its relevance to protein engineering and disease interpretation.^8^ The emergence of Deep Mutational Scanning (DMS)^9^ has greatly expanded the available pool of mutation–stability data sets, driving the development of numerous AI-based methods for predicting mutational effects on stability. Despite their growing popularity, however, these approaches often exhibit limited accuracy, owing to insufficient data in the target application domains as well as systematic biases and imbalances in the training data sets on which they rely.^8,10–14^

Physics-based approaches such as FoldX^15,16^ and Rosetta^17–19^ have proven useful in various high-throughput screening contexts via tools such as MutateX^20^ and RosettaDDGPrediction.^21^ However, depending on the application, these methods can also exhibit suboptimal accuracy, as highlighted in the systematic assessments of protein stability and variant-effect predictors,^12,22^ and, similar to ML-based approaches, they are primarily developed and evaluated for well-structured proteins, with much less established applicability to proteins with substantial flexibility or intrinsic disorder.^23^ Additionally, such approaches typically do not account for the contributions of unfolded states to protein stability.^24,25^ Molecular dynamics (MD)–based methods, particularly enhanced sampling techniques such as replica-exchange molecular dynamics (REMD),^26^ metadynamics,^27^ and alchemical approaches such as thermodynamic integration^28^ have also been explored for this purpose. However, owing to their high computational cost and technical complexity, these methods are primarily applied to relatively small sets of carefully chosen systems and have not yet been widely adopted in high-throughput workflows.

To address these limitations, we explore in this work a novel approach to the engineering of protein and peptide FESs, with particular emphasis on data-scarce regimes^29^ in which large deep-learning (DL) models are often less effective. Our approach builds on the recently developed Collective Variables for Free-Energy Surface Tailoring (CV-FEST) framework,^30,31^ which learns collective variables (CVs) from MD data to capture slow, relevant conformational transitions. CV-FEST is specifically suited to low-throughput settings where only a limited number of FES calculations are feasible.

As an initial proof of concept, we apply our methodology to the folding and unfolding of a short peptide. Peptides provide a convenient testbed: their modest size enables fully atomistic MD simulations across diverse sequences at reasonable computational cost, allowing detailed characterization of thermodynamic, dynamic, and kinetic behavior. At the same time, they capture key principles underlying protein stability and conformational dynamics. Beyond their methodological utility, peptides are of practical interest due to their central roles in biological recognition and regulation,^32^ their growing use as therapeutic modalities,^33–35^ and their intrinsic conformational flexibility, which makes them valuable models for disordered and partially ordered protein systems.^36,37^ Peptides also hold promise for antibacterial applications and responsive biomaterials.^7^

Using the ten-residue β-hairpin CLN025 ^38^ (Figure 1a) as a representative model system, we test whether CV-FEST-derived CVs, learned from short unbiased MD simulations, can (i) identify mutation-sensitive positions in the peptide and (ii) predict shifts in the folding free-energy difference Δ*G* between folded and unfolded basins.

**Figure 1:**
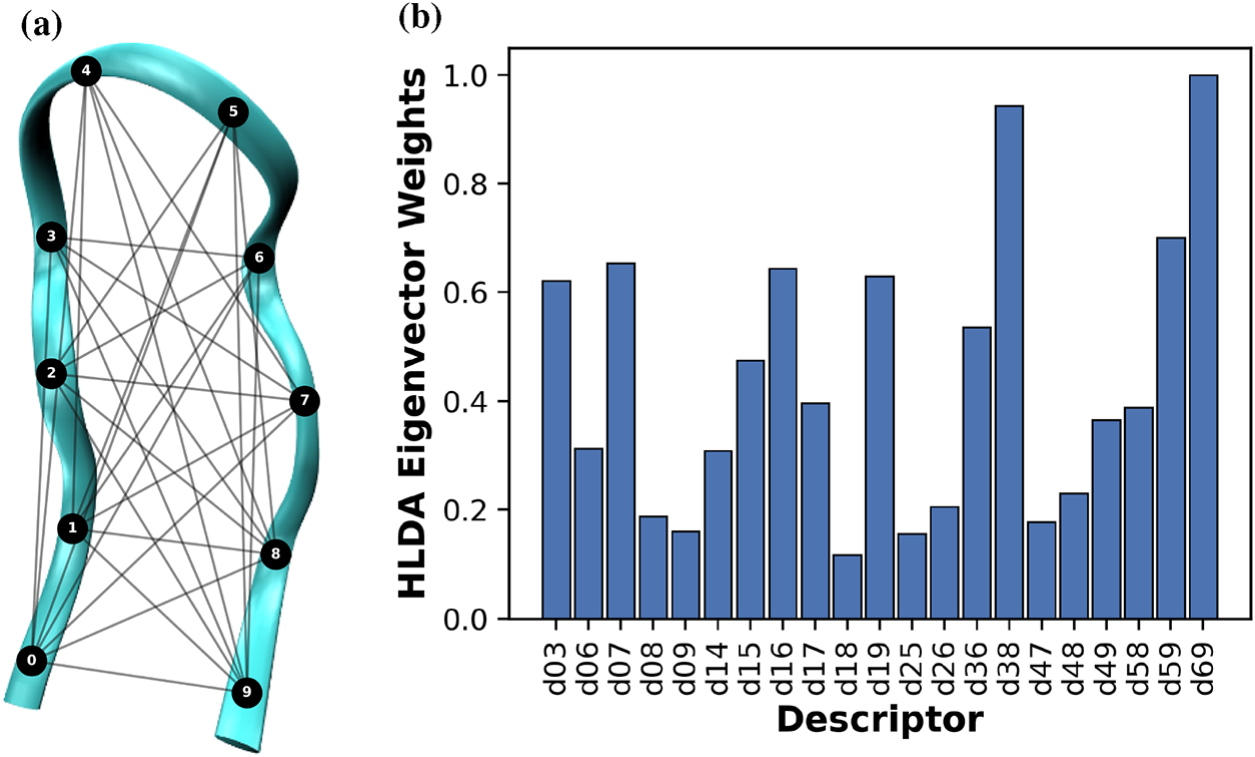
(a) Structural model of CLN025 with inter-residue distance descriptors (black lines). (b) Absolute HLDA eigenvector weights for each distance descriptor.

## Methods

The introduced methodology builds on CV-FEST, a framework we previously developed for free-energy engineering in systems dominated by rare events.^30,31^ CV-FEST was developed to address several practical challenges associated with applying many machine-learning–based approaches to molecular and materials systems, including large data requirements, limited interpretability, and restricted generalizability beyond the training domain. Unlike conventional methods that depend on extensive data sets drawn from many simulations or experiments, which are often unavailable or strongly biased, CV-FEST leverages dynamical information obtained from MD trajectories of a single system or a small set of related systems. The framework is based on the assumption that the essential behavior of slow collective modes can be captured through a low-dimensional representation of the FES defined by an appropriate set of CVs. This compact representation concentrates the most relevant information about the system into a reduced design subspace of possible system modifications, which can be efficiently explored and optimized.^30,31^

CVs constitute a central component of many enhanced sampling techniques,^27,39,40^ guiding the exploration of the system’s phase space and defining the reduced coordinate space in which the corresponding FES is constructed. CVs are functions of the atomic coordinates, **s**(**R**), and their probability distribution is given by

1 $$P\left(s^{\prime }\right)=\int \mathrm{dR}\delta \left[s^{\prime }-s\left(R\right)\right]P\left(R\right)$$

where *P*(**s**′) denotes the probability of observing a given CV value **s**′, *P*(**R**) is the Boltzmann probability distribution, and δ is the Dirac delta function. Using eq 1, the associated FES is then defined as

2 $$F\left(s\right)=-k_{B}T\ln P\left(s\right)$$

where *k*_B_ is the Boltzmann constant and *T* is the temperature.

CVs that enable efficient sampling typically capture the essential physics of a system’s slow processes encoded in the FES defined in eq 2, making them valuable for studying structure–dynamics–function relationships. Although constructing such CVs is challenging as the relationship between molecular structure and the underlying FES tends to be complex, previous work^41–48^ has shown that ML-based approaches can be effective.

### Collective Variables

In the current study, we construct the CVs using Harmonic Linear Discriminant Analysis (HLDA).^41–44,49–51^ HLDA represents each CV as linear combination of user-defined descriptors, which facilitates interpretability: descriptors with larger weights contribute more strongly to the system’s slow collective behavior. In addition, training HLDA is straightforward and data-efficient, requiring only limited sampling within the metastable basins between which the relevant rare events occur.

Mathematically, given a descriptor set, HLDA first estimates the mean vectors **μ** and covariance matrices **Σ**_*I*_ of the descriptors for each metastable state *I*, using short unbiased trajectories of those states. It then seeks a direction **W** in the *N*_*d*_-dimensional descriptor space that maximally separates the projected training distributions by maximizing the Rayleigh quotient

3 $$J\left(W\right)=\frac{W^{T}S_{b}W}{W^{T}S_{w}W}$$

The scatter matrices appearing in eq 3 are defined as follows. For two metastable states *A* and *B*, the between-class scatter matrix is

4 $$S_{b}=\left(\mu_{A}-\mu_{B}\right)(\mu_{A}-\mu_{B}{)}^{T}$$

while the within-class scatter matrix uses the HLDA harmonic average

5 $$S_{w}={\left(\Sigma_{A}^{-1}+\Sigma_{B}^{-1}\right)}^{-1}$$

where **μ**_A,B_ and **Σ**_A,B_ are the means and covariances of states *A* and *B*, respectively, and eqs 4 and 5 define the between-class and within-class contributions to the HLDA objective.

Under the normalization **W** ^*T*^ **S**_*w*_ **W** = 1, maximizing eq 3 is equivalent to solving the generalized eigenvalue problem

6 $$S_{w}^{-1}S_{b}W=\lambda W$$

In eq 6, the leading eigenvector defines the HLDA CV, the direction minimizing overlap between projected folded and unfolded ensembles, while the associated eigenvalue λ quantifies separability along this axis (larger λ indicating better discrimination), a metric we exploit herein.

These eigenvectors assign interpretable weights to the descriptors (Figure 1b), revealing their relative contributions to separability along the CV axis. In previous studies,^30,31^ we showed that targeted modulation of forces associated with highly weighted descriptors enables systematic shifts in the relative thermodynamic stability of the states. Building on this concept, we extend the framework to a more realistic setting by introducing explicit chemical perturbations in the form of point mutations, rather than directly tuning interaction parameters. The resulting eigenvectors and eigenvalues are then used to guide mutation selection and to predict the associated changes in the free-energy difference Δ*G*.

For CV construction, we used inter-residue distance descriptors defined as the distances between the centers of mass of different residues, excluding nearest- and next-nearest-neighbor residue pairs (i.e., only pairs with |*i* – *j*| ≥ 3 are included), yielding a total of 28 descriptors (Figure 1a). To improve numerical stability in the HLDA calculations, we performed correlation-based pruning prior to HLDA by removing highly correlated descriptor pairs (see more details in the Computational Details section). Residue-importance scores were computed from the wild-type (WT) HLDA eigenvector by aggregating descriptor-level contributions at the residue level. After obtaining the leading HLDA eigenvector **W** over the pruned descriptor set (with weights *w*_*ij*_), descriptor weights were assigned to residues by grouping all descriptors involving each residue *r*. The per-residue importance was then defined as the mean absolute weight,

7 $$I_{r}=\frac{1}{2N_{r}}\sum_{\left(i,j\right):i=r\vee j=r}\left|w_{\mathrm{ij}}\right|$$

where the factor of 2 is given that *w*_*ij*_ = *w*_*ji*_, and *N_r_* is the number of descriptors associated with residue *r*. Descriptors removed during pruning were included by assigning them the weight of the retained descriptor to which they were most strongly correlated.

As noted above, because HLDA assigns the largest weights to descriptors most strongly associated with the considered slow mode, modifying the forces linked to these descriptors, e.g., through chemical perturbations such as point mutations, is expected to have the greatest impact on the free-energy difference between the participating metastable states. Because these descriptors also dominate separability along the one-dimensional CV axis, we hypothesized that mutation-induced changes in separability could serve as a surrogate for alterations to the FES governing the rare event. To test this, we use the leading HLDA eigenvalue λ as a measure of folded–unfolded separability and quantify mutation-induced changes relative to the wild type via Δλ = λ_mut_ – λ_WT_, providing a compact metric of how substitutions strengthen or weaken ensemble separation and, by extension, alter the free-energy difference between the metastable basins.

## Computational Details

### Simulation Setup

All MD simulations were performed using GROMACS 2019.6 ^52^ patched with PLUMED 2.9.^53^ For each system WT and mutants, the minimum-enthalpy structure was used as the initial configuration. Systems were modeled using the CHARMM22* force field with the TIP3P water model,^54,55^ solvated in a cubic box containing approximately 1800 water molecules and neutralized with sodium ions. Following energy minimization, systems were equilibrated in the canonical (NVT) ensemble at 340 K using the velocity-rescaling thermostat^56^ with a 2 fs time step. Covalent bonds involving hydrogen atoms were constrained using LINCS,^57^ and long-range electrostatics were treated using the particle-mesh Ewald (PME) method.^58^

### Training Data

For each system, two short unbiased trajectories were generated to provide training data for CV construction: one initiated from a folded, native-like structure and one initiated from an unfolded conformation obtained by temporarily restraining the peptide end-to-end distance prior to release. Each trajectory was propagated for 100 ns at 340 K and used for subsequent analysis, following preliminary assessments that confirmed convergence of the HLDA metrics.

In the short unbiased trajectories used to construct the HLDA training ensembles, spontaneous transitions between folded and unfolded conformations were occasionally observed. To limit mixing between the state-specific ensembles, folded and unfolded states were defined using a root-mean-square deviation (RMSD) CV computed relative to a system-specific minimum-enthalpy reference structure (see Figure S1). Because the resulting ensemble statistics showed some sensitivity to the chosen boundaries, we repeated the analysis over a range of RMSD threshold pairs. The thresholds yielding the strongest WT residue-importance correlations were practically identical to those producing the strongest Δλ–Δ*T*_m_ correlations, considering the former is evaluated on the WT alone, whereas the latter spans both WT and mutants. Importantly, both correlations persist over broad ranges of threshold values (see Figures S2 and S3), with the upper limit of the folded state corresponding to the system’s free-energy barrier region (Figure 2a), and, interestingly, the unfolded-state boundary aligning with RMSD values characteristic of the fully unfolded ensemble while excluding partially folded conformations.

**Figure 2:**
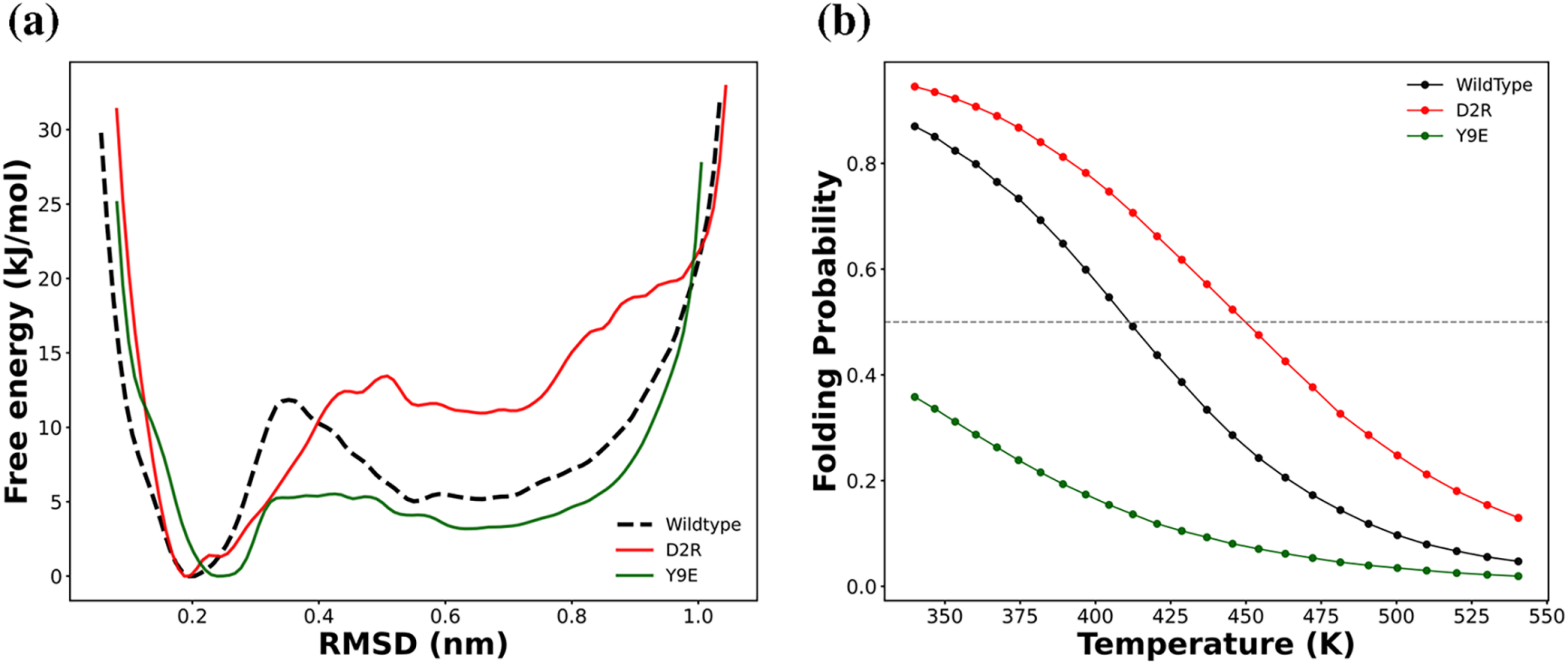
Comparison of WT CLN025 and two representative mutants. (a) Free-energy profiles along the RMSD CV at 340 K showing mutation-dependent changes. (b) Folded probability versus temperature from REMD simulations, with *T*_m_ defined at *P*_folded_ = 0.5.

### Descriptor Set Preprocessing

To improve numerical stability in the HLDA calculations, we performed correlation-based pruning prior to HLDA by removing descriptor pairs with an absolute correlation exceeding a tolerance *r*_tol_. We carried out this pruning separately within the folded and unfolded ensembles using Pearson correlations and then kept only the descriptors that survived both filters. Since eigenvector-derived HLDA metrics are more perturbation-sensitive than eigenvalues,^59^ we applied a stricter tolerance (*r*_tol_ = 0.93) for eigenvector-based analyses than for eigenvalue-based measures (*r*_tol_ = 0.98). This choice further improved the stability and robustness of the results with respect to the definition of state boundaries.

### REMD Simulations and Melting-Temperature Estimations

REMD simulations^26^ were performed in the NVT ensemble using 25 replicas. The replica temperatures were distributed geometrically from 340.0 to 540.5 K by using a scaling factor of *a* = 1.0195, and exchange attempts were made every 1 ps. Conformational states were classified using the RMSD to the native folded reference structure, with RMSD values defining the folded and unfolded basins in accordance with the computed FES (Figure 2a). The first 100 ns of each replica trajectory was discarded for equilibration. For each temperature, we computed the folded-state probability as *P*_folded_(*T*) = *N*_F_/(*N*_F_ + *N*_U_), where *N*_F_ and *N*_U_ are the numbers of frames assigned to the folded and unfolded basins, respectively.^60^ The melting temperature *T*_m_ was defined as *P*_folded_(*T*_m_) = 0.5. Each system was simulated with multiple independent REMD runs; within each run, trajectories were divided into blocks of varying sizes, and *T*_m_ was recomputed for each block to assess convergence. Final *T*_m_ values were obtained by combining runwise estimates, and uncertainties were estimated from the variability across blocks. Replica-exchange mixing and sampling convergence were assessed using standard diagnostics such as temperature-space random walks, RMSD time series for the lowest-temperature replica, and block-size convergence of *T*_m_ (see Figure S9), following the approach in ref 61.

## Results

To assess whether our framework can help guide the selection of point mutations for reshaping the WT FES, we analyzed a systematic mutation scan of the CLN025 peptide and compared CV-derived quantities with thermodynamic benchmarks obtained from REMD simulations. In total, we introduced 36 single-point mutations across seven of the ten residues of the peptide, selected to provide broad coverage along the sequence; the full list of mutant sequences is provided in Table S1. At each site, the native residue was substituted with four to eight amino acids spanning distinct physicochemical classes, including charged, polar, hydrophobic, and aromatic residues, in order to evaluate their effects on the peptide’s FES.

Mutation-induced changes in the FES were characterized using REMD by examining free-energy profiles projected along the RMSD CV, which exhibit pronounced, mutation-dependent variations (Figure 2a). These variations manifest as shifts in the relative free-energy balance between folded and unfolded regions and lead to corresponding changes in the peptide’s *T*_m_, defined by Δ*G*(*T*_m_) = 0 (equivalently *P*_folded_(*T*_m_) = 0.5), as illustrated by the temperature dependence of *P*_folded_ in Figure 2b. To evaluate the ability of the HLDA CV to capture these mutation-induced FES changes, we compare the melting temperatures obtained from REMD with HLDA CV-derived descriptors.

### WT HLDA-Derived Residue-Importance Scores

As a starting point, we first examine whether an HLDA model trained on the WT folded and unfolded ensembles can provide residue-level guidance on mutation sensitivity. To this end, we computed a WT HLDA residue-importance score (eq 7) by aggregating the absolute values of the individual descriptor weights in the HLDA eigenvector (see Figure 1b) associated with each residue, thereby quantifying how strongly each site is, on average, linked to the folded–unfolded transition. The residue-importance scores were then compared with the mean change in the peptide’s melting temperature, Δ*T*_m_ = *T*_m_^mut^ – *T*_m_^WT^, averaged over the different substitutions introduced at each site (i.e., across the set of amino acids used).

We observe a pronounced inverse correlation between these quantities (Figure 3a). This trend suggests that residues that are more prominently represented on average in the HLDA CV tend to correspond to positions where substitutions are associated with a larger destabilization of the folded state. Conversely, the residue with the lowest average HLDA weight was found to exhibit stabilizing substitutions. Across the full set of single mutants, destabilizing variants constitute the majority and dominate the HLDA signal, which is consistent with the general observation that stabilizing mutations are generally harder to capture accurately than destabilizing ones.^13^

**Figure 3:**
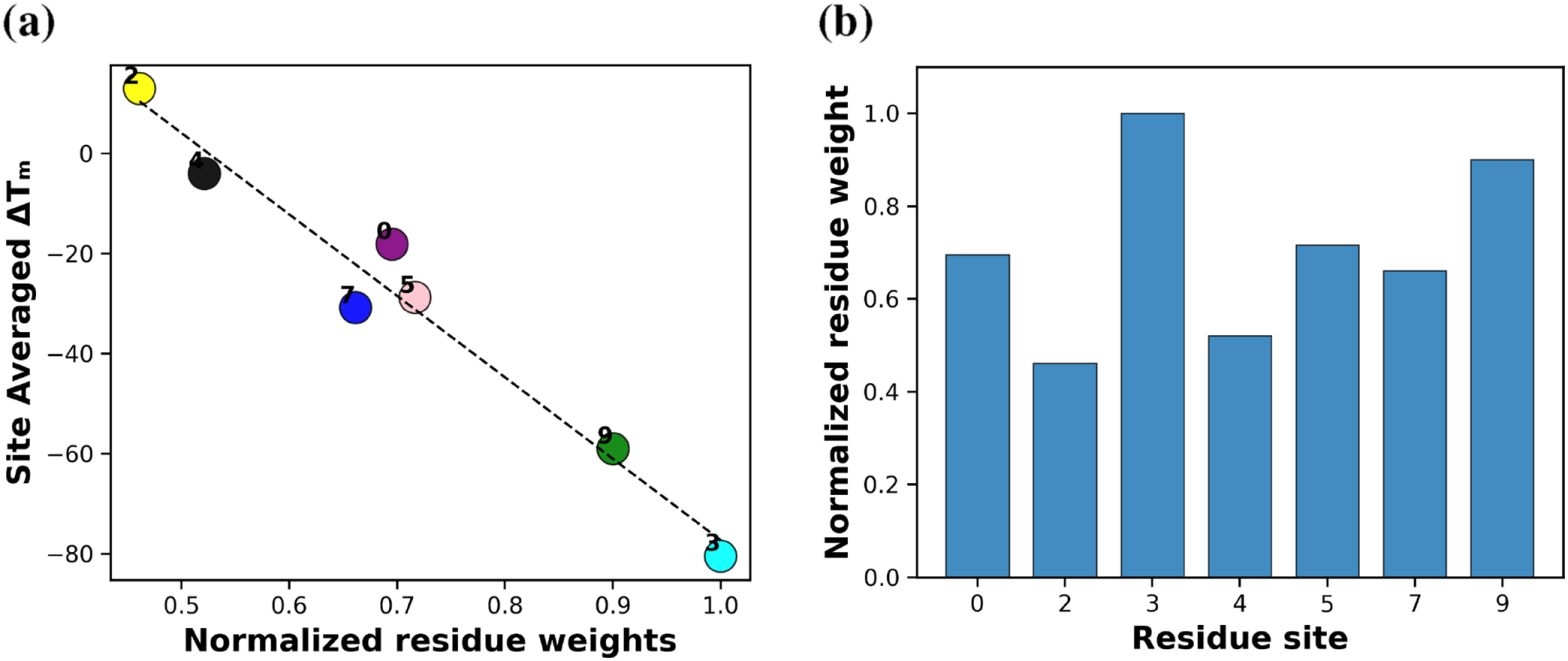
WT HLDA eigenvector-derived residue weights versus per-residue mean changes in the peptide’s melting temperature: (a) Scatter plot summarizing the relationship between the WT residue score computed using eq 7 and the mean Δ*T*_m_ across mutations at each site (Pearson *r* = – 0.98, *p* = 8.09 × 10^–5^). The corresponding analysis using all 36 individual mutant data points, without site averaging, is shown in Figure S11. (b) Per-residue WT weights shown as bars to highlight differences across positions. Residue weights in both panels are shown after normalization by the maximum WT residue weight.

To verify that this relationship is not solely a consequence of averaging over specific substitutions at each residue position, we also examined the corresponding mutation-level relationship using all 36 individual mutant data points (see Figure S11). The association between WT residue importance and Δ*T*_m_ persists and remains robust under subsampling analysis (see Figure S12). The distribution of Δ*T*_m_ values across different substitutions at a given site indicates at the same time that, although certain positions appear more sensitive to perturbation, both the direction and magnitude of the response depend significantly on the specific amino acid introduced.

It is important to note that the strong correlations observed between the computed HLDA eigenvector weights and the mean changes in Δ*T*_m_ were obtained only when the HLDA training set included data from the fully unfolded ensemble while excluding intermediate partially unfolded states (see Figures S2 and S4). We believe this suggests that, for the present analysis, the statistics of the folded state are the dominant factors governing the observed correlations. However, a more definitive understanding of this behavior would require further investigation across additional libraries of systems.

### Mutation-Specific Stability Changes

We next move from site-averaged sensitivity to mutation-specific effects, asking whether the framework can help predict the *T*_*m*_ shift induced by a particular substitution. As noted above, we hypothesized that mutation-induced changes in the separability between the folded and unfolded states along the HLDA CV could serve as a proxy for the corresponding change in the free-energy difference between the two states, *Δ*Δ*G*, relative to the wild type. To test this idea, we recomputed the HLDA CV for each mutant peptide and measured the corresponding change in λ.

Across the full set of mutants, we observe a positive correlation (*r* = 0.66) between Δλ and the mutation-induced stability changes, quantified by the change in the system’s melting temperature Δ*T*_m_ (Figure 4a), suggesting that substitutions which increase folded–unfolded separability along the HLDA coordinate tend to be stabilizing, whereas those that decrease separability tend to be destabilizing. To further assess whether the leading HLDA directions remain comparable across mutants, we computed the angles between leading HLDA eigenvectors across the mutant panel; this analysis showed that most leading HLDA directions remain broadly aligned across sequences (see Figure S13).

**Figure 4:**
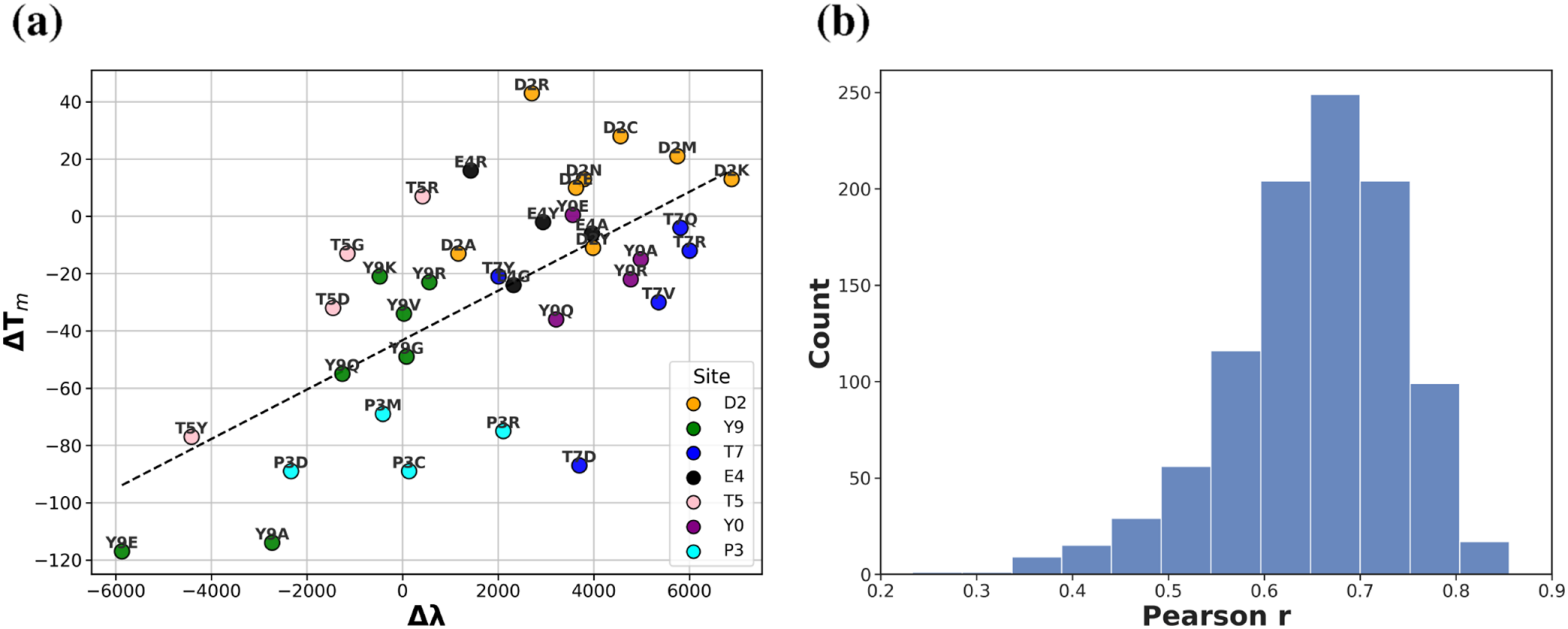
Mutation-level relationship between HLDA eigenvalue shifts and changes in the peptide thermodynamic conformational stability: (a) Correlation between Δ*λ* and Δ*T*_m_ (Pearson *r* = 0.66, *p* = 1.1 × 10^–5^; Spearman ρ = 0.61, *p* = 7.6 × 10^–5^). (b) Subsampling analysis demonstrating the robustness of the correlation upon excluding a fraction of mutants.

Interestingly, we find that removing the two most prominent outliers in the data set (D2R and T7D) increases the Pearson correlation up to *r* = 0.77 (see Figures S5 and S6). We were able to identify a relatively straightforward explanation for the outlier behavior of D2R (see Section S6 and Figure S7). In contrast, despite further analysis, we were unable to identify a similarly simple explanation for the deviation observed for T7D, including the possibility that it arises from the presence of a misfolded state. Additional folded-basin free-energy projections for selected mutants are provided in Section S6 (Figure S8).

To further assess the robustness of this relationship, we performed a subsampling analysis in which the correlation was recomputed 100 times after randomly removing 40% of the mutants. The resulting distribution of correlation coefficients is centered at *r* = 0.66, indicating that the observed trend is robust to data subsampling (Figure 4b).

We also compared the predictions of our approach against a physicochemical analysis based on the chemical categories of the amino acids involved in each mutation (see Figure S10). Within the mutant library considered in this study, it was difficult to identify clear predictive trends based on these classifications alone. While changes in residue charge appeared to show some indication of correlation with Δ*T*_m_, this observation should be interpreted with caution given the limited amount of data available for such an analysis. Concomitantly, and subject to the same limitations, we did not observe a substantial correlation (*r* ∼ 0.4) between the predictions of our method and the physicochemical properties considered. This could suggest that the information captured by our approach extends beyond the specific mutation descriptors examined here.

## Discussion and Conclusions

Tuning the free-energy surface (FES) of biomolecules such as peptides and proteins through point mutations is a highly complex task that is combinatorially intractable. Even for a peptide consisting of only 10 residues, a brute-force approach would require the computation of the FES for 20^10^ possible mutants, making direct computation of mutation-induced free-energy changes impractical. Building on the CV-FEST framework, the goal of this study was to use collective variables (CVs) to guide targeted chemical modifications that systematically reshape the free-energy landscape while avoiding prohibitive computational cost.

Specifically, we employed HLDA to construct guiding CVs, motivated by its simplicity, interpretability, and ability to be trained using relatively small amounts of data obtained from short unbiased simulations performed within the system’s metastable states. The attained results indicate that this approach can lead to a significant reduction in the computational cost. In particular, a CV constructed for the wild-type (WT) system provides residue-level information that appears to be informative of whether replacement of a given residue is more likely to stabilize or destabilize the peptide’s folded state. This behavior is reflected in the observed correspondence between the WT HLDA CV weights and the average change in the peptide melting temperature as estimated from REMD simulations (Figure 3).

Beyond providing guidance on which residues may be most sensitive to mutation, we further find that the proposed methodology can also assist in informing the choice of amino acid substitutions at a selected position. This is achieved by constructing HLDA CVs for individual mutants and comparing the leading HLDA eigenvalue of each mutant to that of the WT system, thereby quantifying changes in the separability of the two basins along the one-dimensional HLDA coordinate. Although this quantity does not represent a direct estimate of the free-energy difference, here too we observe that variations in the leading HLDA eigenvalue are consistent with trends in the mutation-induced shift of the free-energy balance between the basins (Figure 4). This suggests that the eigenvalue difference may serve as a practical and computationally inexpensive indicator of the relative stabilization or destabilization. A related application of this framework has also shown that mutation-specific HLDA eigenvalues correlate with peptide unfolding kinetics.^62^

Unlike many machine-learning–based approaches to stability prediction that rely on large experimental data sets, a key advantage of the proposed methodology is that it requires no extensive training data. Instead, it relies solely on readily accessible and computationally inexpensive information obtained from short unbiased simulations confined to the metastable states of the systems of interest, thereby capturing intrinsic thermal fluctuations within those states, a concept similarly exploited by interaction-centric tools such as Key Interactions Finder (KIF).^63^ An important takeaway is that even short trajectories sampled locally within the relevant conformational basins can contain predictive information about the free-energy difference between them, even in the absence of observed transitions. Because the approach is grounded in a physical framework, it may also provide mechanistic insights into the molecular factors governing changes in the FES, a direction which we intend to explore in future work.

As shown, during the development of the methodology, we did observe some dependence on data preprocessing, for example, in determining the precise boundaries used to define the states from which the unbiased training data are extracted. While a uniform definition offers a consistent reference across mutants, point mutations may alter state boundaries and transition thresholds, indicating that more-system-specific definitions could be beneficial. In the long term, we envision several possible remedies, including the use of more systematic and automated state-identification procedures that can be adjusted for each mutant, employing alternative descriptors to the RMSD for defining the state boundaries as well as extending the framework to incorporate more advanced deep-learning architectures for CV construction. In the near term, a practical approach could be to refine state boundaries using a small validation set of mutants for which full REMD calculations are carried out and then applying the resulting calibrated cutoffs to support efficient exploration of a broader mutation space. To further evaluate the robustness and generality of the approach, future work will extend its application to larger and more complex peptides and proteins.

## Supplementary Material



## Data Availability

All scripts and input files required to reproduce the analyses in this study are publicly available at github/MendelsResearchGroup/CV-guided-FES-engineering. The repository contains Python scripts for HLDA analysis, threshold scanning, correlation analysis, and figure generation, together with the GROMACS and PLUMED input files used in this work. It also includes the GROMACS structure and topology files for all systems analyzed in the study, including the corresponding .gro, .top, and .itp files. Molecular dynamics trajectories are not included due to their size but can be provided by the authors upon reasonable request.

## Notes

List of single-point mutant sequences analyzed in this study (Section S1); details on RMSD-based state-boundary selection used to define folded and unfolded ensembles for HLDA (Section S2); robustness of the HLDA-based correlations to the choice of RMSD thresholds (Section S3); threshold dependence of the WT residue-importance analysis (Section S4); robustness of the Δλ–Δ*T*_m_ correlation to outlier removal (Section S5); outlier analysis for D2R and T7D (Section S6); REMD convergence diagnostics supporting the reliability of the melting-temperature benchmarks (Section S7); physicochemical analysis of mutation effects (Section S8); individual mutant analysis of WT residue importance versus mutation-specific Δ*T*_m_ (Section S9); and HLDA eigenvector direction-angle analysis across the mutant panel (Section S10) (PDF)
