# Supplementary material for: Collective Variable-Guided Engineering of the Free-Energy Surface of a Small Peptide
Source: J Chem Inf Model. 2026 Jul 10;66(14):8465–73. doi: 10.1021/acs.jcim.6c00674 (PMC13418176; doi:10.1021/acs.jcim.6c00674)
Supplement: Supplementary file 1 [file ci6c00674_si_001.pdf]

Supporting Information

Collective Variable-Guided Engineering of the Free-Energy Surface of a Small Peptide

Muralika Medaparambath[1,2], Alexander Zhilkin[1], Dan Mendels*[1]

[1]The Wolfson Department of Chemical Engineering, Technion – Israel Institute of Technology, Haifa 32000, Israel

[2]Faculty of Mathematics, Technion – Israel Institute of Technology, Haifa 32000, Israel

Contents of the Supporting Information

S.1. Single-point mutant sequences
S.2. RMSD-based state-boundary selection
S.3. RMSD-threshold robustness for HLDA-based correlations
S.4. Threshold dependence of the WT residue-importance analysis
S.5. Robustness of the $\Delta\lambda$–ΔT_m correlation to outlier removal
S.6. Outlier analysis for D2R and T7D
S.7. REMD convergence analysis
S.8. Physicochemical analysis of mutation effects
S.9. Individual-mutant analysis of WT residue importance versus ΔT_m
S.10. HLDA eigenvector direction-angle analysis

S.1. Single-point mutant sequences

Table S1. Single-point mutants analyzed in this study. Mutation sites are numbered according to the residue indexing used in Fig. 4a of the main text. The wild-type CLN025 sequence is `YYDPETGTWY`. Each sequence differs from the wild type by one residue substitution only.

No.	Mutant	Mutation site	WT residue	Mutant residue	Peptide sequence
1	Y0A	0	Y	A	`AYDPETGTWY`
2	Y0E	0	Y	E	`EYDPETGTWY`
3	Y0Q	0	Y	Q	`QYDPETGTWY`
4	Y0R	0	Y	R	`RYDPETGTWY`
5	D2A	2	D	A	`YYAPETGTWY`
6	D2C	2	D	C	`YYCPETGTWY`
7	D2F	2	D	F	`YYFPETGTWY`
8	D2K	2	D	K	`YYKPETGTWY`
9	D2M	2	D	M	`YYMPETGTWY`
10	D2N	2	D	N	`YYNPETGTWY`
11	D2R	2	D	R	`YYRPETGTWY`

Continued on next page

Table S1 – continued from previous page

No.	Mutant	Mutation site	WT residue	Mutant residue	Peptide sequence
12	D2Y	2	D	Y	YYYPETGTWY
13	P3C	3	P	C	YYDCETGTWY
14	P3D	3	P	D	YYDDETGTWY
15	P3M	3	P	M	YYDMETGTWY
16	P3R	3	P	R	YYDRETGTWY
17	E4G	4	E	G	YYDPGTGTWY
18	E4K	4	E	K	YYDPKTGTWY
19	E4R	4	E	R	YYDPRTGTWY
20	E4Y	4	E	Y	YYDPYTGTWY
21	T5D	5	T	D	YYDPEDGTWY
22	T5G	5	T	G	YYDPEGGTWY
23	T5R	5	T	R	YYDPERGTWY
24	T5Y	5	T	Y	YYDPEYGTWY
25	T7D	7	T	D	YYDPETGDWY
26	T7Q	7	T	Q	YYDPETGQWY
27	T7R	7	T	R	YYDPETGRWY
28	T7V	7	T	V	YYDPETGVWY
29	T7Y	7	T	Y	YYDPETGYWY
30	Y9A	9	Y	A	YYDPETGTWA
31	Y9E	9	Y	E	YYDPETGTWE
32	Y9G	9	Y	G	YYDPETGTWG
33	Y9K	9	Y	K	YYDPETGTWK
34	Y9Q	9	Y	Q	YYDPETGTWQ
35	Y9R	9	Y	R	YYDPETGTWR
36	Y9V	9	Y	V	YYDPETGTWV

S.2. RMSD-based state-boundary selection

Fig. S1 shows RMSD time series for two short unbiased trajectories initialized from folded and unfolded conformations. RMSD was computed relative to the system-specific minimum-enthalpy folded reference structure. Dashed lines indicate a representative folded/unfolded threshold pair used for ensemble labeling, matching the main-text $\Delta\lambda$–ΔT_m analysis. Robustness to threshold choice is evaluated via the threshold scans in Figs. S2 and S3.

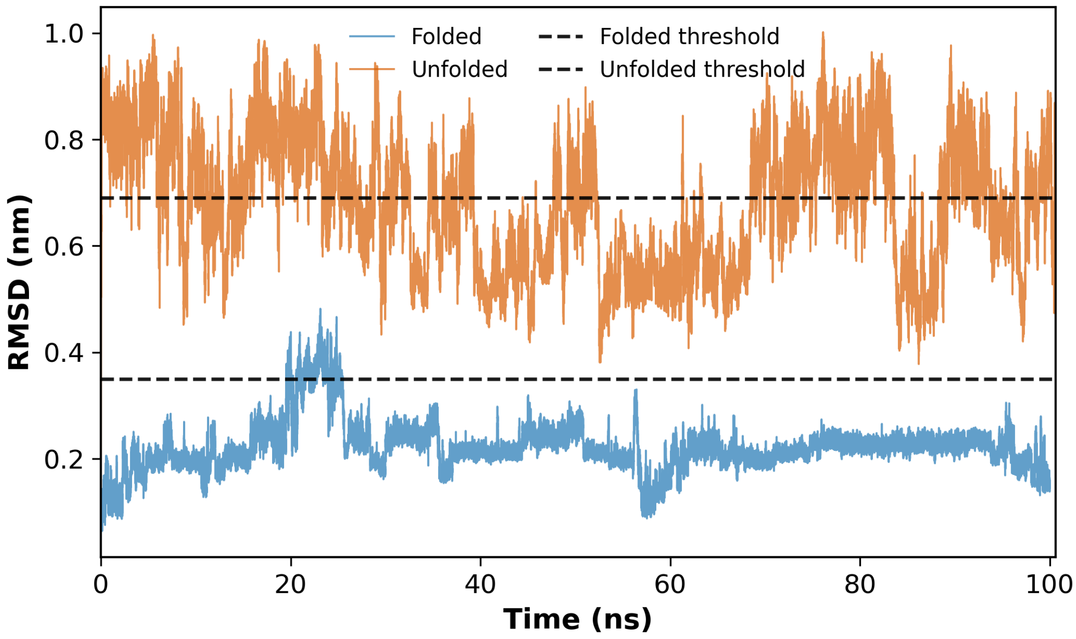

Fig. S1. RMSD versus time for the folded-start (blue) and unfolded-start (orange) unbiased trajectories. Dashed lines denote the representative RMSD thresholds used here ($\mathrm{thr}_F = 0.35$ nm, $\mathrm{thr}_U = 0.69$ nm), corresponding to the main-text $\Delta\lambda$–ΔT_m analysis.

S.3. RMSD-threshold robustness for HLDA-based correlations

Figs. S2 and S3 quantify how the reported correlations depend on the RMSD thresholds used to define folded and unfolded ensembles.

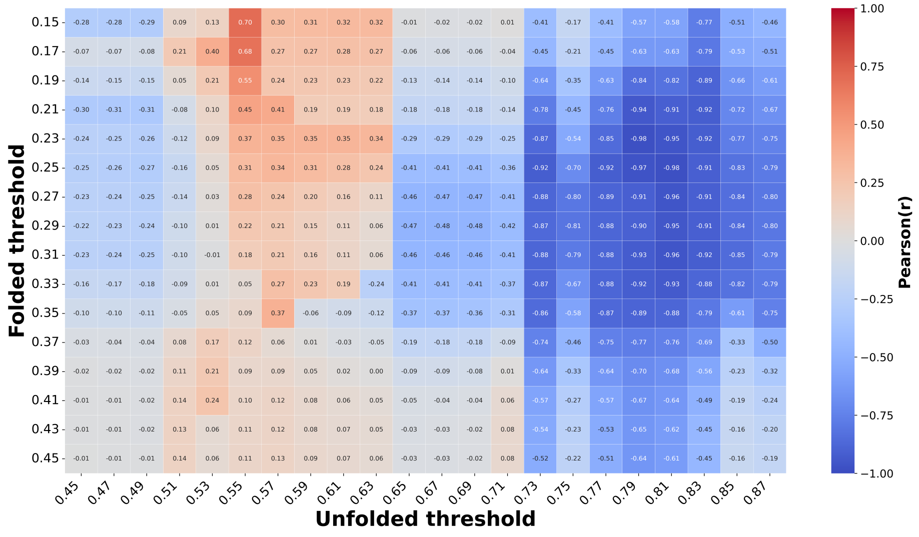

Fig. S2. Pearson correlation heatmap between WT HLDA residue importance and average signed ΔT_m across threshold pairs.

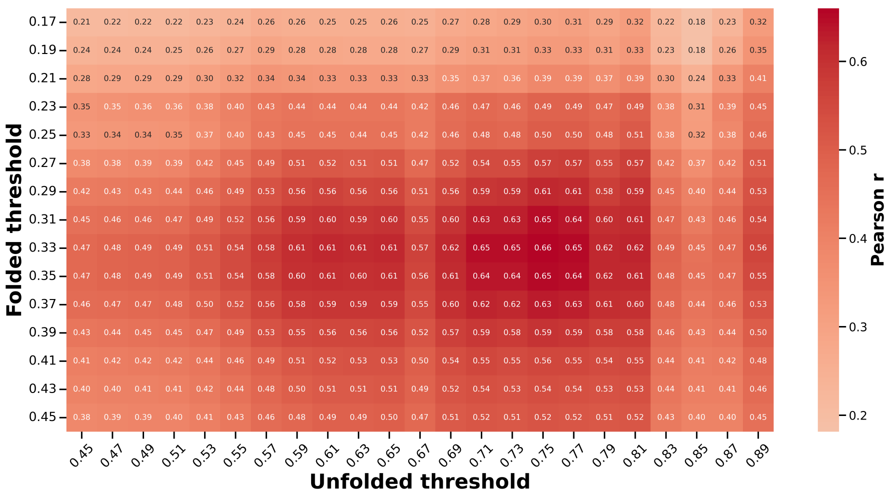

Fig. S3. Pearson correlation between $\Delta\lambda$ and ΔT_m across folded/unfolded RMSD thresholds.

S.4. Threshold dependence of the WT residue-importance analysis

To better understand the threshold dependence observed for the WT residue-importance correlation in Fig. S2, we examined WT unfolded-start trajectories under different unfolded-state threshold definitions. For visualization, selected configurations were projected onto the two highest-weighted HLDA distance descriptors, d_{06} and d_{38}, where d_{ij} denotes the inter-residue distance descriptor between residues i and j in the 0-indexed descriptor notation used for the HLDA analysis.

As shown in Fig. S4, overly restrictive unfolded-state thresholds include too little unfolded-state data, whereas lower thresholds incorporate intermediate partially unfolded structures into the unfolded ensemble. The strongest WT residue-importance correlations are obtained in the intermediate regime, where the selected configurations primarily correspond to the fully unfolded ensemble. These observations suggest that, for the present analysis, the relevant HLDA statistics are best captured when the folded ensemble is compared against a sufficiently sampled fully unfolded ensemble, while excluding intermediate partially unfolded states.

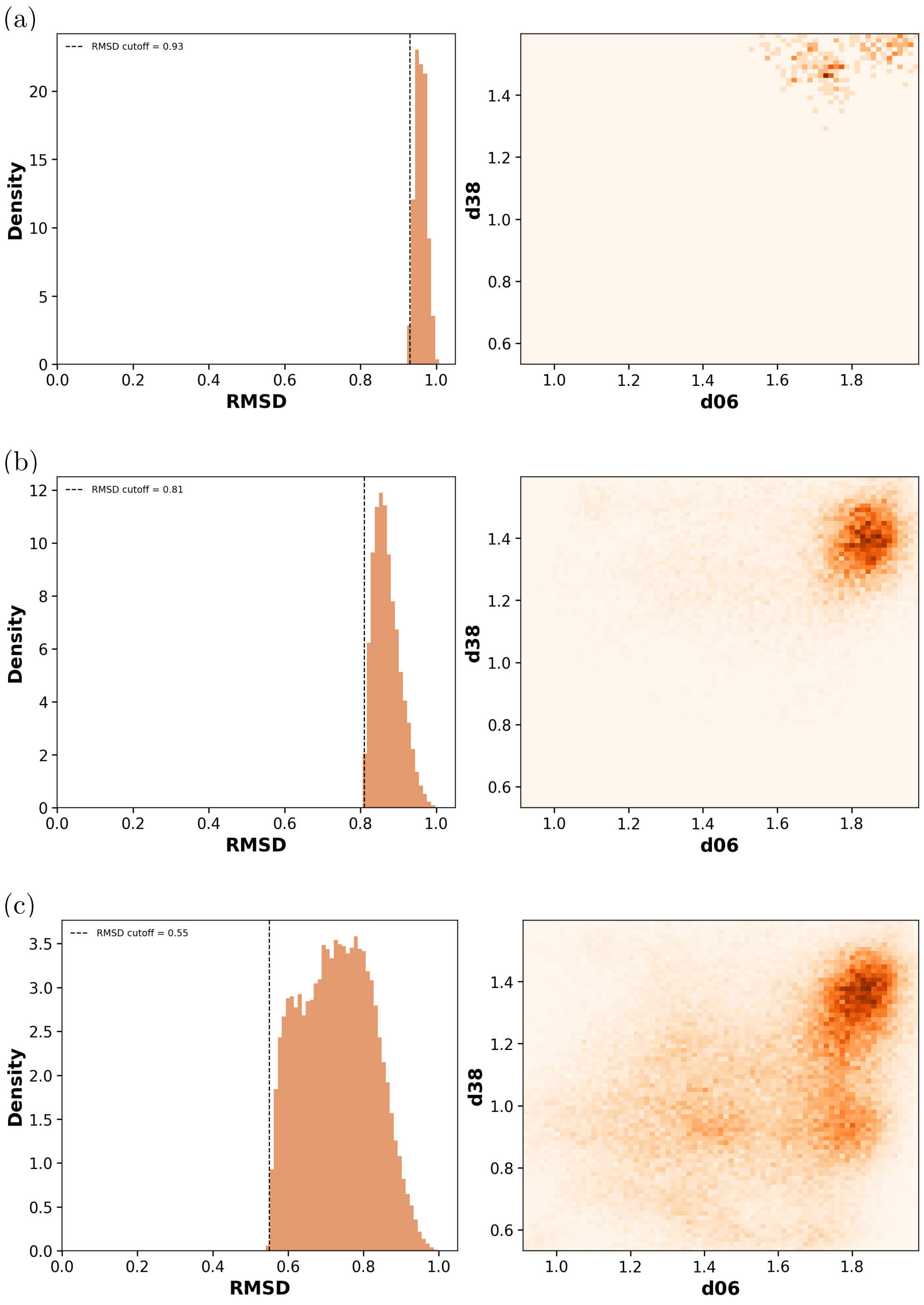

Fig. S4. Representative WT unfolded-basin data distributions under different unfolded-state threshold definitions, projected onto the two highest-weighted HLDA distance descriptors, d_{06} and d_{38}. Here, d_{ij} denotes the inter-residue distance between residues i and j in the 0-indexed residue notation. (a) Restrictive unfolded-state definition with limited unfolded-state data. (b) Threshold choice in which the selected configurations primarily correspond to the fully unfolded ensemble. (c) Lower unfolded-state threshold in which intermediate partially unfolded structures are increasingly included. Dashed lines indicate the predefined unfolded-state boundary used in each case.

S.5. Robustness of the $\Delta\lambda$–ΔT_m correlation to outlier removal

To check whether the observed positive $\Delta\lambda$–ΔT_m trend is dominated by two extreme mutants, we repeated the analysis after removing D2R and T7D from the full 36-mutant data set. For the threshold pair shown in Fig. S5, removal of these two mutants gave a Pearson correlation of $r = 0.77$ ($p = 8.6 \times 10^{-8}$) and a Spearman correlation of $\rho = 0.74$ ($p = 6.32 \times 10^{-7}$). This indicates that the positive $\Delta\lambda$–ΔT_m relationship is not driven solely by these two extreme cases.

The corresponding threshold-sweep heatmap in Fig. S6 shows the same analysis across folded/unfolded RMSD threshold choices. After removal of D2R and T7D, the $\Delta\lambda$–ΔT_m correlation persists across a broad range of threshold combinations, indicating that the positive relationship is not restricted to a single threshold choice.

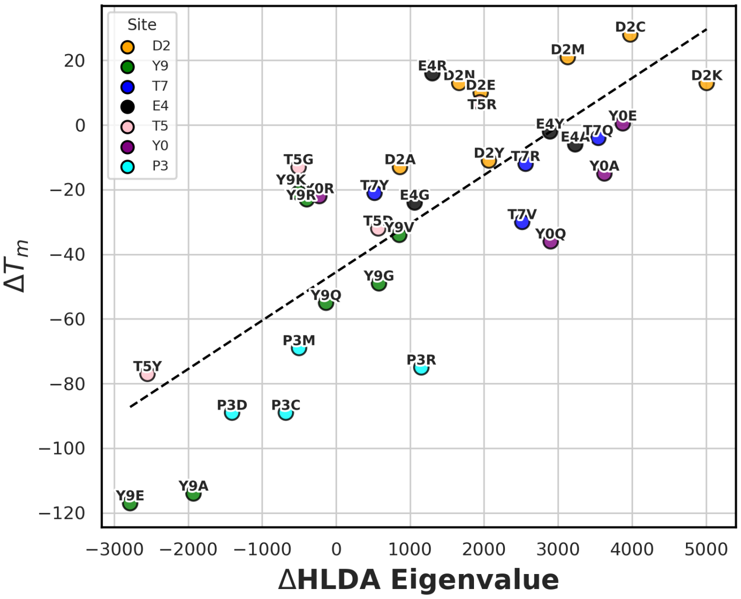

Fig. S5. Scatter plot of $\Delta\lambda$ versus ΔT_m after removing D2R and T7D from the data set. For this filtered 34-mutant data set, the Pearson correlation is $r = 0.77$ ($p = 8.6 \times 10^{-8}$) and the Spearman correlation is $\rho = 0.74$ ($p = 6.32 \times 10^{-7}$).

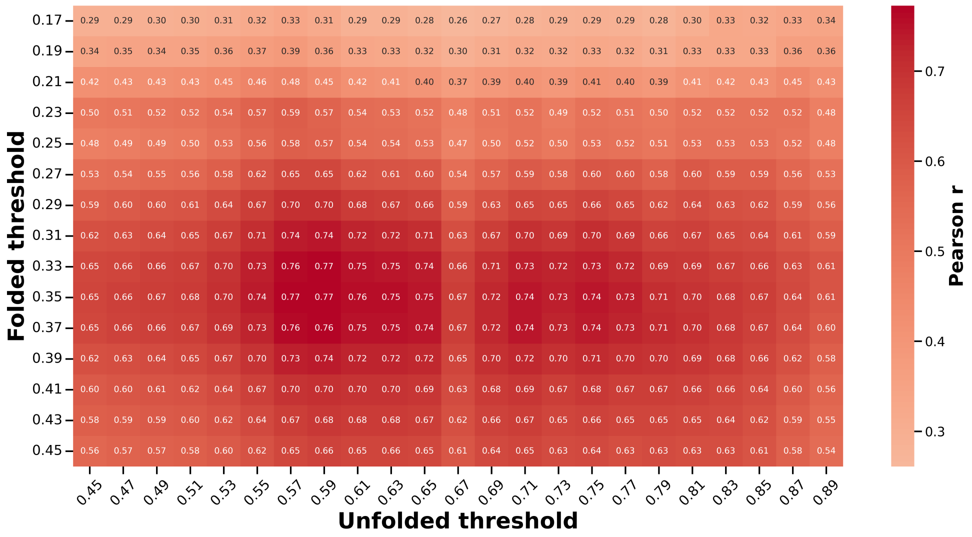

Fig. S6. Threshold-sweep heatmap of the Pearson correlation between $\Delta\lambda$ and ΔT_m after removal of D2R and T7D.

S.6. Outlier analysis for D2R and T7D

The two largest deviations in the $\Delta\lambda$–ΔT_m analysis correspond to D2R and T7D. After removing these two mutants, the correlation increases to approximately $r \approx 0.74$–0.77, depending on the folded/unfolded state-boundary definitions used. We therefore examined whether the outlier behavior of these mutants could be related to differences in their underlying free-energy profiles.

For D2R, the free-energy profile shows a clear shift in the barrier region relative to WT. This suggests that the metastable-state boundaries for D2R are not well aligned with the common state-boundary definitions used throughout the mutant set in the $\Delta\lambda$ analysis in Fig. 4, providing a plausible explanation for its outlier behavior. In contrast, T7D does not show an analogous shift in this RMSD-projected free-energy profile, indicating that state-boundary misalignment does not explain its deviation in the same way.

We additionally examined the folded-basin free-energy surfaces in the plane defined by the Asp3N–Gly7O and Asp3N–Thr8O distances, which have been used to distinguish between the folded and misfolded states of CLN025[1]. While we observed a possible indication of a misfolded minimum for Y9A, we did not detect similar behavior for the mutants that appear as outliers in Fig. 4(a) of the main text, including T7D. This suggests that their deviation from the overall trend is unlikely to originate from the presence of a hidden misfolded state.

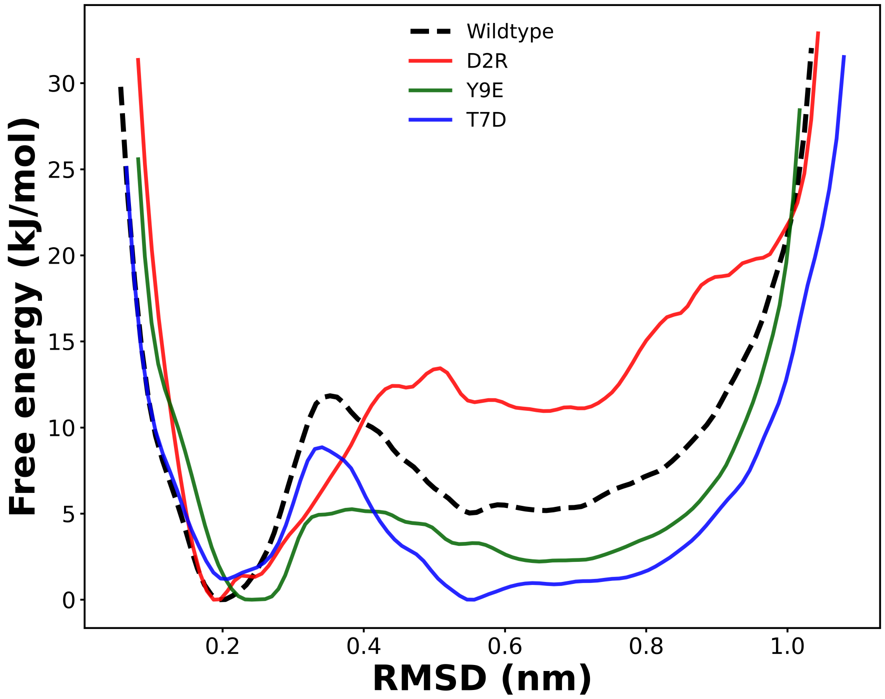

Fig. S7. RMSD-projected free-energy profiles for WT and selected mutants at 340 K. D2R and T7D are shown as the two largest outliers in the $\Delta\lambda$–ΔT_m analysis, and Y9E is shown as an additional mutant reference.

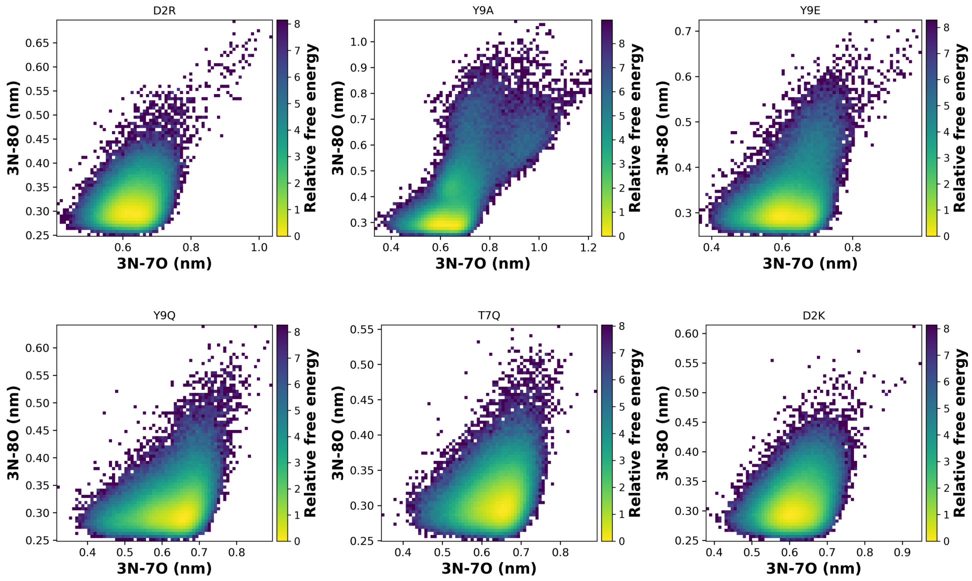

Fig. S8. Folded-basin free-energy surfaces in the Asp3N–Gly7O / Asp3N–Thr8O plane for selected mutants. These coordinates are known to distinguish folded and misfolded states of CLN025[1].

S.7. REMD convergence analysis

To assess convergence and reliability of the REMD benchmarks, we evaluated (i) temperature-space mixing, (ii) structural stability over time, and (iii) block-size stability of thermodynamic estimates.

Fig. S9 shows these diagnostics for a representative system: a temperature trace and RMSD time series for the lowest-temperature replica, together with a block analysis of T_m.

The block analysis was performed using multiple independent REMD trajectories, with an aggregate sampling time exceeding 2 μs. After discarding the first 150 ns of each trajectory as equilibration, the remaining data were partitioned into contiguous blocks of 50, 100, 150, 200, and 250 ns.

For each block, $p_{\text{fold}}(T)$ was computed across the 25-replica temperature ladder, and T_m was obtained by interpolation at $p_{\text{fold}} = 0.5$. Block-wise estimates were pooled across runs to report a weighted mean and weighted standard error (SE).

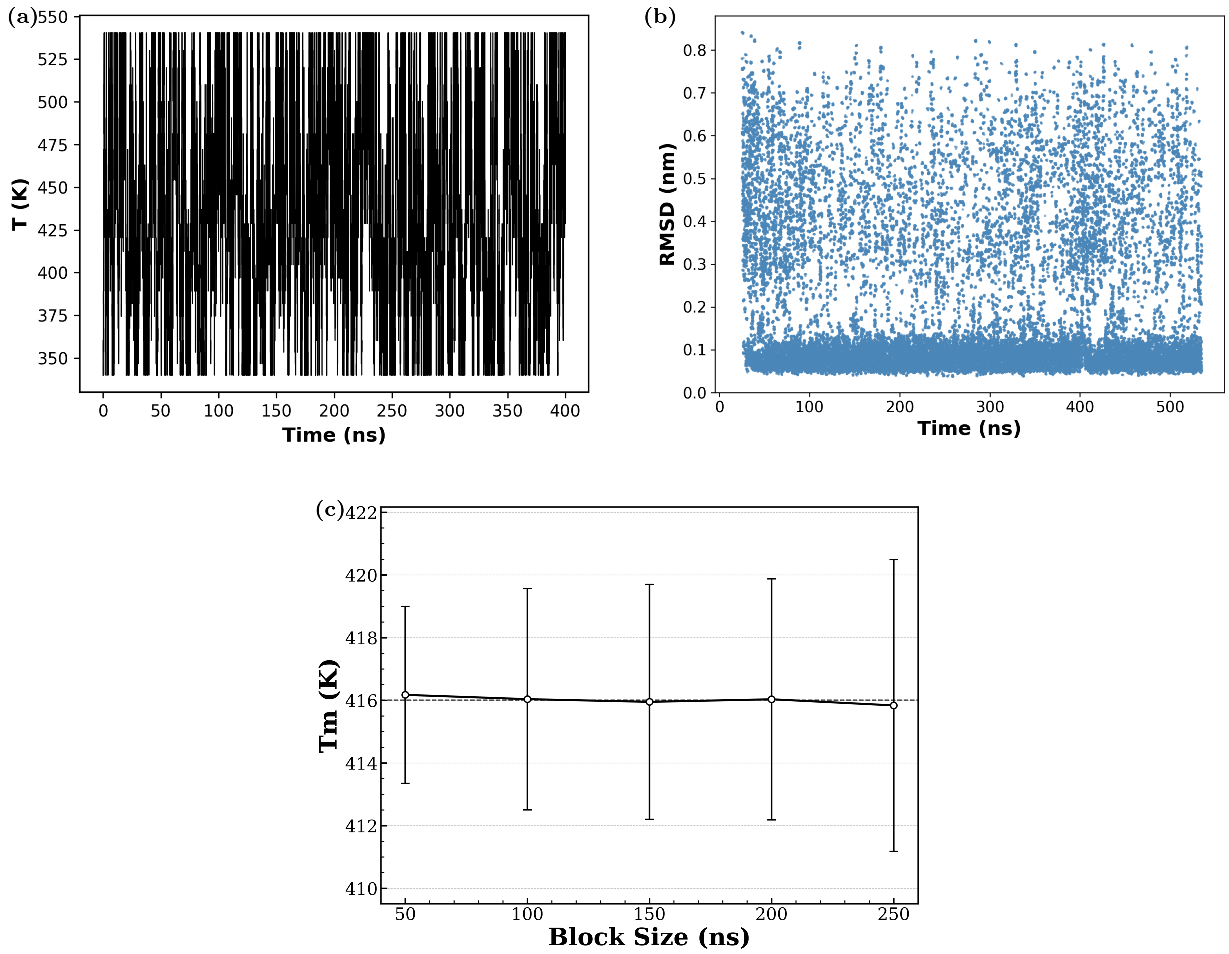

Fig. S9. REMD convergence diagnostics. (a) Temperature-space trace of the low-temperature replica over time. (b) RMSD versus time for the lowest-temperature replica. (c) Block-size convergence of T_m from multiple independent REMD runs of a representative system. Points show weighted mean T_m, error bars show weighted SE for block sizes 50–250 ns, and the dashed line indicates the overall mean.

S.8. Physicochemical analysis of mutation effects

To examine whether simple physicochemical properties of the substitutions are related to the observed melting-temperature shifts, we compared ΔT_m with three mutation-level quantities: change in residue charge, Grantham distance[2], and change in Kyte–Doolittle hydropathy[3]. Fig. S10 shows the corresponding scatter plots for the full 36-mutant data set. The charge-change analysis shows the clearest association with ΔT_m, whereas Grantham distance and hydropathy change do not show clear correlations. Grantham distance and hydropathy change do not show clear correlations with ΔT_m, whereas the change in amino acid charge shows some degree of association. This charge-based trend should, however, be interpreted cautiously, as two of the Δcharge categories contain only a small number of mutations and substantial within-class variability is observed, particularly for Δcharge $= -1$ and Δcharge $= 0$. Nevertheless, these results suggest that charge change may provide useful predictive information, particularly when combined with additional descriptors.

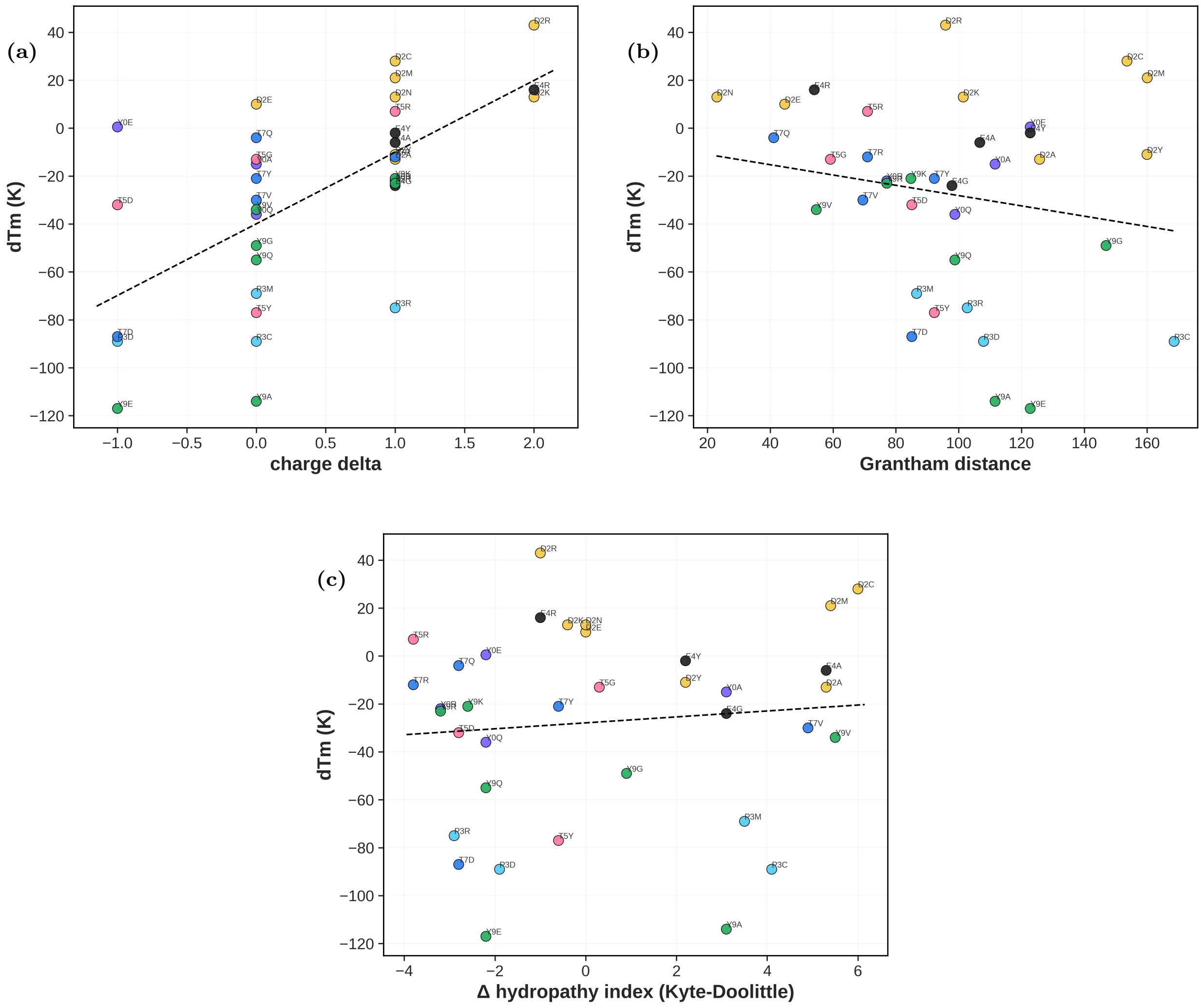

Fig. S10. Physicochemical analysis of mutation-induced melting-temperature shifts. (a) ΔT_m versus change in residue charge between the WT and mutant amino acids. (b) ΔT_m versus Grantham distance between the wild-type and mutant residues. (c) ΔT_m versus change in Kyte–Doolittle hydropathy.

S.9. Individual-mutant analysis of WT residue importance versus ΔT_m

To address the possibility that the site-averaged ΔT_m values in Fig. 3 of the main text depend strongly on the particular substitutions included at each residue position, we also examined the relationship using all 36 individual mutant data points without site averaging. In this analysis, each point corresponds to a single mutant. The x-coordinate is the WT residue importance score of the mutated site, and the y-coordinate is the mutation-specific melting-temperature shift, $\Delta T_m = T_m(\text{mut}) - T_m(\text{WT})$. As shown in Fig. S11, the individual-mutant data retain a strong negative correlation between WT residue importance and ΔT_m (Pearson $r = -0.77$, $p = 5.37 \times 10^{-8}$). With the exception of a few outliers, mutations at the same residue position tend to cluster together, supporting the site-level trend shown in Fig. 3 of the main text.

To further test whether this relationship depends strongly on the particular substitutions included at each site, we performed subsampling analyses over 10,000 iterations. In each iteration, one substitution was randomly omitted from each mutated residue position. We then recomputed the correlation either after recalculating the site-averaged ΔT_m values or using the remaining individual mutant data points directly. In both cases, the correlations remain strongly negative, indicating that the observed relationship is not driven by a small number of specific substitutions.

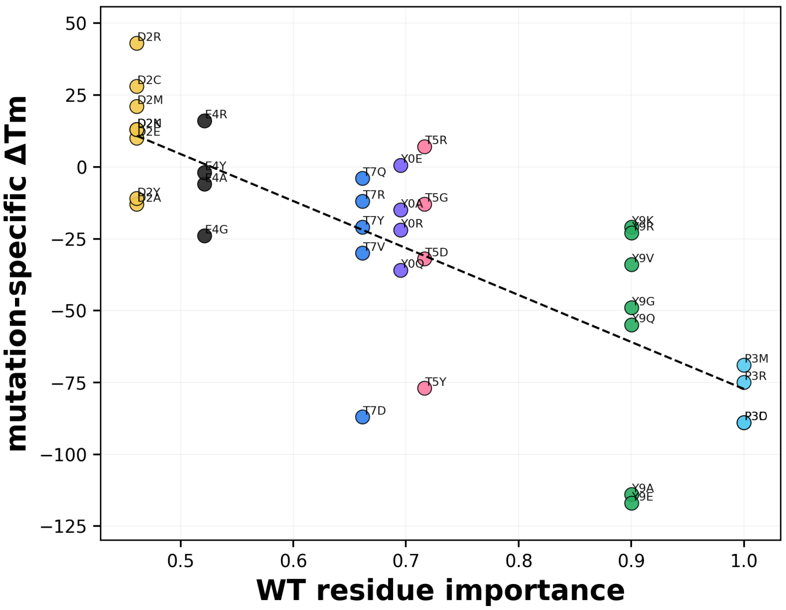

Fig. S11. Individual-mutant relationship between WT residue importance and mutation-specific ΔT_m. Each point represents one of the 36 single-point mutants, without averaging over substitutions at the same residue position. The x-axis shows the WT residue importance score of the mutated site, and the y-axis shows the corresponding mutation-specific $\Delta T_m = T_m(\text{mut}) - T_m(\text{WT})$. The individual-mutant data retain a strong negative correlation (Pearson $r = -0.77$, $p = 5.37 \times 10^{-8}$), indicating that the relationship shown in Fig. 3 of the main text is not solely a consequence of site averaging.

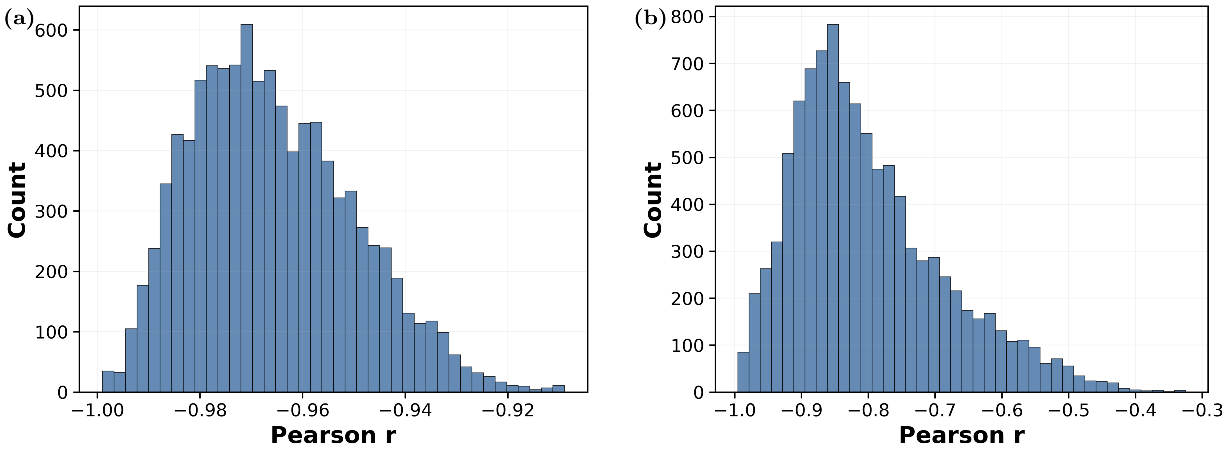

Fig. S12. Subsampling analysis of the relationship between WT residue importance and ΔT_m. In each iteration, one substitution was omitted from each mutated residue position. (a) Distribution of Pearson correlations obtained after recalculating the site-averaged ΔT_m values. (b) Distribution of Pearson correlations obtained using the remaining individual mutant data points directly.

S.10. HLDA eigenvector direction-angle analysis

To assess whether the leading HLDA directions remain similar across all mutants in the library, we calculated the angles between the leading HLDA eigenvectors. The vectors were normalized to unit length before comparison, and the angle between sequences i and j was computed as

$$\theta_{ij} = \arccos\left(\frac{|\mathbf{w}_i \cdot \mathbf{w}_j|}{\|\mathbf{w}_i\|\|\mathbf{w}_j\|}\right),$$

where $\mathbf{w}_i$ and $\mathbf{w}_j$ are the corresponding leading HLDA eigenvectors. The absolute value accounts for the arbitrary sign of the eigenvector.

The angles between the WT eigenvector and each mutant eigenvector showed no meaningful correlation with melting temperature, with Pearson $r = -0.043$, $p_{value} = 0.805$, and Spearman $\rho = -0.197$, $p_{value} = 0.251$ (see Fig. S13a). We also computed the angle distribution over all 666 unique pairwise comparisons among WT and the 36 mutants (see Fig. S13b).

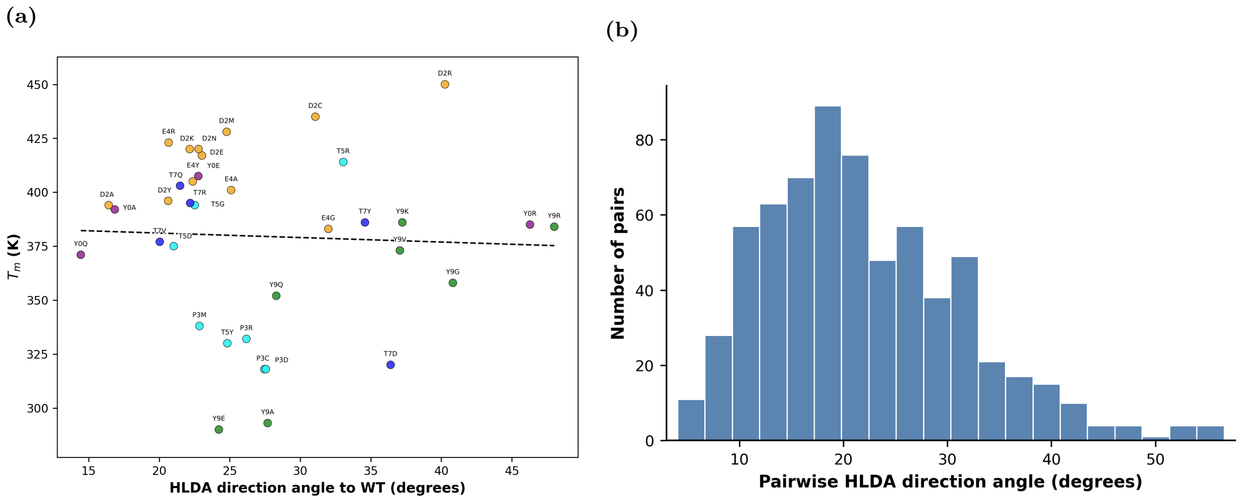

Fig. S13. HLDA eigenvector direction-angle analysis. (a) Angle between the WT leading HLDA eigenvector and each mutant leading HLDA eigenvector, plotted against the corresponding melting temperature. The dashed line indicates a linear fit. (b) Distribution of pairwise direction angles between leading HLDA eigenvectors over all unique sequence pairs among WT and the 36 mutants. Angles were computed after normalizing the leading HLDA eigenvectors to unit length.

References

[1] Lindahl, V.; Lidmar, J.; Hess, B. Accelerated weight histogram method for exploring free energy landscapes. *The Journal of Chemical Physics* **2014**, *141*, 044110.

[2] Grantham, R. Amino Acid Difference Formula to Help Explain Protein Evolution. *Science* **1974**, *185*, 862–864.

[3] Kyte, J.; Doolittle, R. F. A Simple Method for Displaying the Hydropathic Character of a Protein. *Journal of Molecular Biology* **1982**, *157*, 105–132.
